# Supplementary material for: Generic injuries are sufficient to induce ectopic Wnt organizers in Hydra
Source: eLife. 2021 Mar 29;10:e60562. doi: 10.7554/eLife.60562 (PMC8049744; doi:10.7554/eLife.60562)
Supplement: Figure 1—source data 2. — The sequence, shortened name, and full name for all transcription factor binding motifs (TFBMs) that were associated with significant changes in chromatin accessibility during the first 12 hr of head and foot regeneration. All TFBMs were pulled from the list of HOMER motifs provided by the chromVARmotifs package. [file elife-60562-fig1-data2.docx]

| **Motif Sequence** | **Short Name** | **Full Name** |
| --- | --- | --- |
| 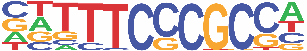 | **E2F7** | **E2F7(E2F)/Hela-E2F7-ChIP-Seq(GSE32673)/Homer** |
| 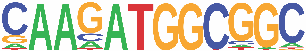 | **YY1** | **YY1(Zf)/Promoter/Homer** |
| 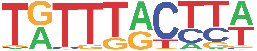 | **FOXM1** | **FOXM1(Forkhead)/MCF7-FOXM1-ChIP-Seq(GSE72977)/Homer** |
| 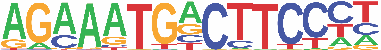 | **ZNF528** | **ZNF528(Zf)/HEK293-ZNF528.GFP-ChIP-Seq(GSE58341)/Homer** |
| 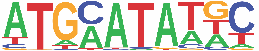 | **Pit1** | **Pit1(Homeobox)/GCrat-Pit1-ChIP-Seq(GSE58009)/Homer** |
| 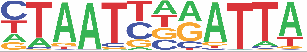 | **Phox2a** | **Phox2a(Homeobox)/Neuron-Phox2a-ChIP-Seq(GSE31456)/Homer** |
| 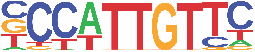 | **Sox2** | **Sox2(HMG)/mES-Sox2-ChIP-Seq(GSE11431)/Homer** |
| 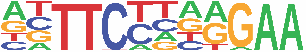 | **STAT6** | **STAT6(Stat)/CD4-Stat6-ChIP-Seq(GSE22104)/Homer** |
| 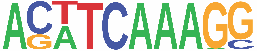 | **TCFL2** | **TCFL2(HMG)/K562-TCF7L2-ChIP-Seq(GSE29196)/Homer** |
| 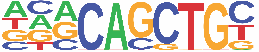 | **E2A** | **E2A(bHLH)/proBcell-E2A-ChIP-Seq(GSE21978)/Homer** |
| 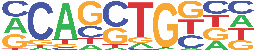 | **HEB** | **HEB(bHLH)/mES-Heb-ChIP-Seq(GSE53233)/Homer** |
| 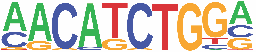 | **ZBTB18** | **ZBTB18(Zf)/HEK293-ZBTB18.GFP-ChIP-Seq(GSE58341)/Homer** |
| 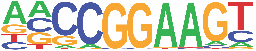 | **ELF1** | **ELF1(ETS)/Jurkat-ELF1-ChIP-Seq(SRA014231)/Homer** |
| 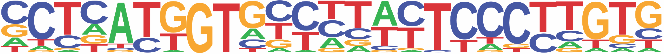 | **ZNF41** | **ZNF41(Zf)/HEK293-ZNF41.GFP-ChIP-Seq(GSE58341)/Homer** |
| 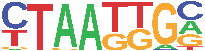 | **Isl1** | **Isl1(Homeobox)/Neuron-Isl1-ChIP-Seq(GSE31456)/Homer** |
| 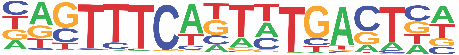 | **bZIP:IRF** | **bZIP:IRF(bZIP,IRF)/Th17-BatF-ChIP-Seq(GSE39756)/Homer** |
| 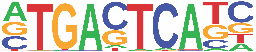 | **AP-1 (TRE)** | **AP-1(bZIP)/ThioMac-PU.1-ChIP-Seq(GSE21512)/Homer** |
| 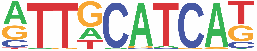 | **Chop (c/EBP)** | **Chop(bZIP)/MEF-Chop-ChIP-Seq(GSE35681)/Homer** |
| 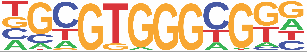 | **Egr2** | **Egr2(Zf)/Thymocytes-Egr2-ChIP-Seq(GSE34254)/Homer** |
| 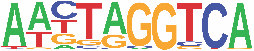 | **RORgt** | **RORgt(NR)/EL4-RORgt.Flag-ChIP-Seq(GSE56019)/Homer** |
| 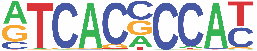 | **Srebp1a** | **Srebp1a(bHLH)/HepG2-Srebp1a-ChIP-Seq(GSE31477)/Homer** |
| 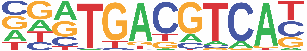 | **Atf7 (CRE)** | **Atf7(bZIP)/3T3L1-Atf7-ChIP-Seq(GSE56872)/Homer** |
| 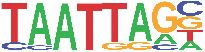 | **Lhx2** | **Lhx2(Homeobox)/HFSC-Lhx2-ChIP-Seq(GSE48068)/Homer** |
| 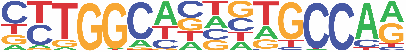 | **NF1** | **NF1(CTF)/LNCAP-NF1-ChIP-Seq(Unpublished)/Homer** |
| 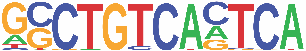 | **PBX1** | **PBX1(Homeobox)/MCF7-PBX1-ChIP-Seq(GSE28007)/Homer** |
| 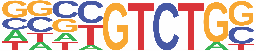 | **Smad4** | **Smad4(MAD)/ESC-SMAD4-ChIP-Seq(GSE29422)/Homer** |
| 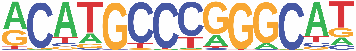 | **P53** | **p53(p53)/mES-cMyc-ChIP-Seq(GSE11431)/Homer** |
| 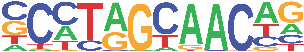 | **Rfx5** | **Rfx5(HTH)/GM12878-Rfx5-ChIP-Seq(GSE31477)/Homer** |
| 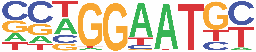 | **TEAD4** | **TEAD4(TEA)/Tropoblast-Tead4-ChIP-Seq(GSE37350)/Homer** |
| 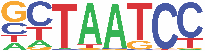 | **CRX** | **CRX(Homeobox)/Retina-Crx-ChIP-Seq(GSE20012)/Homer** |
| 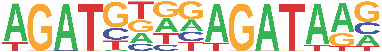 | **GATA3** | **GATA3(Zf),DR4/iTreg-Gata3-ChIP-Seq(GSE20898)/Homer** |
| 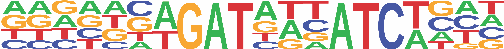 | **GATA** | **GATA(Zf),IR3/iTreg-Gata3-ChIP-Seq(GSE20898)/Homer** |
