# Supplementary material for: Generic injuries are sufficient to induce ectopic Wnt organizers in Hydra
Source: eLife. 2021 Mar 29;10:e60562. doi: 10.7554/eLife.60562 (PMC8049744; doi:10.7554/eLife.60562)
Supplement: Transparent reporting form [file elife-60562-transrepform.docx]

***eLife’s* transparent reporting form**

We encourage authors to provide detailed information *within their submission* to facilitate the interpretation and replication of experiments. Authors can upload supporting documentation to indicate the use of appropriate reporting guidelines for health-related research (see [EQUATOR Network](http://www.equator-network.org/%20)), life science research (see the [BioSharing Information Resource](https://biosharing.org/)), or the [ARRIVE guidelines](http://www.plosbiology.org/article/info:doi/10.1371/journal.pbio.1000412) for reporting work involving animal research. Where applicable, authors should refer to any relevant reporting standards documents in this form.

If you have any questions, please consult our Journal Policies and/or contact us: [editorial@elifesciences.org](mailto:editorial@elifesciences.org).

**Sample-size estimation**

- You should state whether an appropriate sample size was computed when the study was being designed
- You should state the statistical method of sample size computation and any required assumptions
- If no explicit power analysis was used, you should describe how you decided what sample (replicate) size (number) to use

Please outline where this information can be found within the submission (e.g., sections or figure legends), or explain why this information doesn’t apply to your submission:

Information on sample-size estimation for ATAC-seq, RNA-seq, tissue manipulation, and qPCR experiments is provided in the methods sections “ATAC-seq Library Preparation”, “RNA-seq Library Preparation”, “*Hydra* Tissue Manipulation Experiments”, and “Quantitative Reverse Transcription PCR (qPCR)” respectively.

**Replicates**

- You should report how often each experiment was performed
- You should include a definition of biological versus technical replication
- The data obtained should be provided and sufficient information should be provided to indicate the number of independent biological and/or technical replicates
- If you encountered any outliers, you should describe how these were handled
- Criteria for exclusion/inclusion of data should be clearly stated
- High-throughput sequence data should be uploaded before submission, with a private link for reviewers provided (these are available from both GEO and ArrayExpress)

Please outline where this information can be found within the submission (e.g., sections or figure legends), or explain why this information doesn’t apply to your submission:

Information on RNA-seq and ATAC-seq biological replicates is provided in Tables 1 & 2 and the methods sections “ATAC-seq Library Preparation” and “RNA-seq Library Preparation.” Information on qPCR biological replicates is provided in the methods section “Quantitative Reverse Transcription PCR (qPCR)”. Information on tissue manipulation biological replicates is provided in the methods section “*Hydra* Tissue Manipulation Experiments”, the Figure 5 legend, and the Figure 5–Figure Supplement 1 legend. FASTQ files of raw ATAC-seq and RNA-seq reads, expression matrices for ATAC-seq and RNA-seq reads mapped to the Hydra 2.0 genome reference, consensus peak files, and bigwig genome tracks of individual and pooled ATAC-seq replicates are available through the Gene Expression Omnibus under the accession GSE152994.

**Statistical reporting**

- Statistical analysis methods should be described and justified
- Raw data should be presented in figures whenever informative to do so (typically when N per group is less than 10)
- For each experiment, you should identify the statistical tests used, exact values of N, definitions of center, methods of multiple test correction, and dispersion and precision measures (e.g., mean, median, SD, SEM, confidence intervals; and, for the major substantive results, a measure of effect size (e.g., Pearson's r, Cohen's d)
- Report exact p-values wherever possible alongside the summary statistics and 95% confidence intervals. These should be reported for all key questions and not only when the p-value is less than 0.05.

Please outline where this information can be found within the submission (e.g., sections or figure legends), or explain why this information doesn’t apply to your submission:

Statistical analysis methods for ATAC-seq, RNA-seq, qPCR, and tissue manipulation experiments are described in the methods sections “Differential Accessibility and ChromVar Analysis”, “RNA-seq Data Processing and Differential Gene Expression Analysis”, “Quantitative Reverse Transcription PCR (qPCR)”, and “*Hydra* Tissue Manipulation Experiments” respectively. Additionally, full results tables for ATAC-seq and RNA-seq analyses are provided in Supplemental Files 1 & 2 respectively. Full Results for the motif enrichment and motif accessibility analyses are provided in Supplemental File 3.

(For large datasets, or papers with a very large number of statistical tests, you may upload a single table file with tests, Ns, etc., with reference to sections in the manuscript.)

**Group allocation**

- Indicate how samples were allocated into experimental groups (in the case of clinical studies, please specify allocation to treatment method); if randomization was used, please also state if restricted randomization was applied
- Indicate if masking was used during group allocation, data collection and/or data analysis

Please outline where this information can be found within the submission (e.g., sections or figure legends), or explain why this information doesn’t apply to your submission:

Information on group allocation does not apply to our submission as all animals used for different experimental groups were drawn from the same clonally propagated culture.

**Additional data files (“source data”)**

- We encourage you to upload relevant additional data files, such as numerical data that are represented as a graph in a figure, or as a summary table
- Where provided, these should be in the most useful format, and they can be uploaded as “Source data” files linked to a main figure or table
- Include model definition files including the full list of parameters used
- Include code used for data analysis (e.g., R, MatLab)
- Avoid stating that data files are “available upon request”

Please indicate the figures or tables for which source data files have been provided:

All code used in this study is available both as a git repository at github.com/cejuliano/jcazet_regeneration_patterning and on Dryad at doi.org/10.25338/B8S612. Source data are provided for plots in Figure 1C-I, Figure 2A-D, Figure 2 – Figure Supplement 1, Figure 3C-H, and Figure 3 – Figure Supplement 2E,F. In addition, we provide full ATAC-seq and RNA-seq differential test results tables generated by EdgeR in Supplemental Files 1 & 2 respectively. We also provide motif enrichment statistics for injury responsive peaks generated by HOMER and motif chromatin accessibility variability scores generated by chromVAR in Supplemental file 3.
